# Cerebral Vascular Disturbances Following Traumatic Brain Injury: Pathophysiology, Diagnosis, and Therapeutic Perspectives—A Narrative Review

**DOI:** 10.3390/life15091470

**Published:** 2025-09-18

**Authors:** Nicoleta-Larisa Serban, Gheorghe Ungureanu, Ioan Stefan Florian, Daniela Ionescu

**Affiliations:** 1 Department of Neurosurgery, Cluj County Clinical Emergency Hospital, 400347 Cluj-Napoca, Romania; stefanfloriannch@gmail.com; 2 Department of Neurosciences, “Iuliu Hatieganu” University of Medicine and Pharmacy, 400012 Cluj-Napoca, Romania; 3 Department of Anesthesiology and Intensive Care, “Iuliu Hatieganu” University of Medicine and Pharmacy, 400162 Cluj-Napoca, Romania; 4 Outcome Research Consortium, Cleveland, OH 44195, USA

**Keywords:** traumatic brain injury, cerebrovascular dysfunction, cerebral blood flow, cerebral autoregulation, cerebral ischemia, cerebrovascular resistance, endothelial dysfunction

## Abstract

Traumatic brain injury (TBI) is a major global health concern and a leading cause of long-term disability and mortality. While the primary mechanical insult is often the focus of acute care, secondary injury mechanisms—particularly cerebrovascular dysfunction—play a critical role in ongoing neural damage and poor outcomes. Increasing research highlights the role of neurovascular changes in TBI pathophysiology. This narrative review compiles evidence from the past decade on mechanisms, diagnostic methods, and treatments related to cerebrovascular dysfunction after TBI. A structured search of PubMed and Embase identified relevant clinical and preclinical studies. Key mechanisms include blood–brain barrier disruption, impaired cerebral autoregulation, microthrombosis, and oxidative stress. Diagnostic tools discussed include perfusion imaging, cerebrovascular reactivity testing, and blood-based biomarkers of vascular injury. Therapeutic strategies targeting the neurovascular unit are categorized by mechanism: anti-inflammatory agents (e.g., celecoxib, minocycline), mitochondrial protectors (e.g., Tanshinone IIA), and vasomodulators (e.g., sildenafil). We propose an integrated therapeutic approach for a multimodal treatment plan that integrates these interventions. The findings emphasize the importance of patient-specific vascular therapies to reduce secondary ischemic injury and enhance neurological recovery. Although promising preclinical data exist, clinical application remains limited. More well-designed trials are needed to confirm the safety and effectiveness of emerging therapies.

## 1. Introduction

Traumatic brain injury (TBI) remains a primary global health concern. According to the Global Burden of Disease 2021 study, there were approximately 20.8 million incident cases of TBI and 37.9 million prevalent cases worldwide, resulting in about 5.5 million years lived with disability (YLDs) [1]. Although advances in acute neurocritical care have improved survival, many patients continue to experience long-term neurological and cognitive impairments, emotional disturbances, and an elevated risk of developing neurodegenerative diseases [2,3].

Historically, TBI research and clinical management have focused mainly on the primary mechanical injury to brain parenchyma. However, emerging evidence increasingly highlights the role of secondary cerebrovascular disturbances as key factors in ongoing brain damage and poor outcomes [4,5]. These vascular impairments include microvascular disruption, hypoperfusion, blood–brain barrier (BBB) breakdown, impaired cerebral autoregulation, and microthrombi formation, all of which contribute to metabolic crises, neuroinflammation, and neuronal death [6,7,8]. Recent studies also suggest that even mild TBI can lead to subtle yet persistent microvascular dysfunction detectable through advanced imaging or biomarker analysis [9,10].

Despite their clinical significance, cerebrovascular consequences of traumatic brain injury are frequently underrecognized and underreported. Given that many of these vascular alterations develop over hours to days, a more comprehensive understanding of the underlying secondary pathophysiology may facilitate the identification of novel diagnostic biomarkers, reveal potential therapeutic targets, and determine optimal treatment windows [11]. Imaging techniques such as cerebral perfusion imaging, arterial spin labeling, Xenon-CT, and advanced MRI methods have enabled the measurement of cerebral blood flow (CBF), cerebrovascular reactivity (CVR), and BBB integrity in both experimental models and human patients [12,13,14].

The identification of circulating biomarkers, such as ischemia-modified albumin (IMA), NSE, and inflammatory ratios (NLR, PLR), offers potential for the early detection of vascular impairment and cerebral ischemia [4,5,6]. Novel therapies targeting the vascular compartment—from improved physiological management (e.g., THE MANTLE bundle) to experimental agents such as Tanshinone IIA—are promising in preclinical and early clinical studies [2,6,7].

This study aims to systematically evaluate the pathophysiological impact and clinical relevance of cerebrovascular alterations in TBI. To this end, we conducted a targeted literature search of PubMed and Embase, focusing on peer-reviewed articles published over the past decade. The review includes both clinical and preclinical studies investigating the mechanisms of cerebrovascular dysfunction, diagnostic modalities, and novel therapeutic interventions.

The objective was to synthesize current knowledge on cerebrovascular disturbances following TBI, following three essential aspects:

1. The pathophysiological mechanisms underlying vascular injury and dysfunction.
2. Diagnostic tools and biomarkers used for detecting and monitoring cerebrovascular compromise.
3. Emerging and experimental therapeutic strategies targeting the vascular system to improve clinical outcomes.

By integrating evidence from both basic science and clinical research, this review aims to clarify the neurovascular unit’s role in secondary brain injury progression and to identify opportunities for timely, targeted interventions during the acute and subacute phases of TBI.

## 2. Materials and Methods

### 2.1. Search Strategy and Databases

A structured literature search was conducted to identify studies published between January 2014 and December 2024. The main databases used were PubMed and Embase, which yielded 498 records from PubMed and 325 records from Embase (total = 823). To minimize the risk of missing relevant studies, the Cochrane Library and Web of Science were also screened, but no additional eligible articles were retrieved.

The search strategy combined the MeSH term *“traumatic brain injury”* with Boolean operators and relevant keywords. An example of the search string used in PubMed is as follows:

(“traumatic brain injury” [MeSH Terms] OR “traumatic brain injury” [Title/Abstract] OR “TBI” [Title/Abstract]) AND (“cerebral blood flow” [Title/Abstract] OR “blood–brain barrier” [Title/Abstract] OR “microvascular dysfunction” [Title/Abstract] OR “cerebrovascular autoregulation” [Title/Abstract] OR “perfusion” [Title/Abstract] OR “neuroinflammation” [Title/Abstract] OR “therapeutic strategies” [Title/Abstract] OR “vascular-targeted therapy” [Title/Abstract])

Equivalent Entree terms were used for Embase. The last search was performed in December 2024.

**Figure 1:**
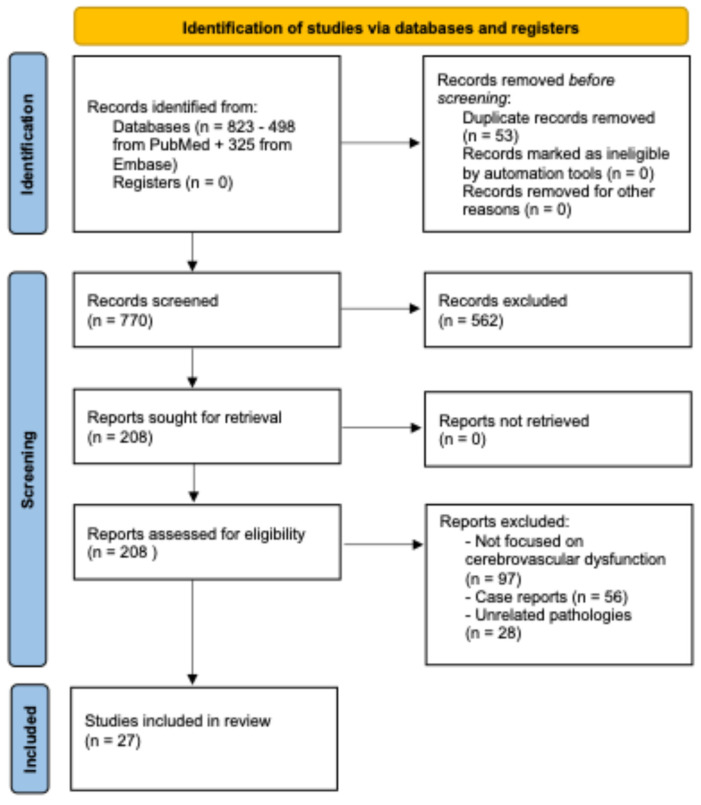
Flow diagram of the literature search and study selection process.

The complete selection process following the search is described in Section 2.2 and illustrated in Figure 1.

### 2.2. Study Selection and Eligibility Criteria

The initial database search yielded 823 results (PubMed = 498, Embase = 325). After removal of duplicates (n = 53), a total of 770 articles remained. These were screened in two stages:

1. Title and Abstract Screening: A total of 770 studies were assessed. Based on predefined inclusion/exclusion criteria, 208 articles were retained for full-text evaluation.
2. Full-Text Review: After applying exclusion criteria (non-original studies, editorials, case reports, non-cerebrovascular focus, or unrelated pathologies), 27 studies were selected for final inclusion.

After removal of duplicates (**n = 53**), a total of **770 records** remained. These were screened in two stages:

1. Title and abstract screening: 770 articles were assessed, and **562 were excluded** for being unrelated to cerebrovascular dysfunction in TBI.
2. **Full-text review:** 208 full-text articles were evaluated. Of these, **181 were excluded** because they were non-original studies (reviews, editorials, commentaries), case reports, or conference abstracts, or did not focus primarily on cerebrovascular dysfunction.

Following this process, **27 studies** met all criteria and were included in the qualitative synthesis, as illustrated in Figure 1.

### 2.3. Data Extraction and Categorization

Selected studies were categorized into three main thematic domains:

1. **Pathophysiology**—microvascular injury, BBB disruption, autoregulatory failure.
2. **Diagnostics**—imaging techniques, perfusion monitoring, biomarkers.
3. **Therapeutics**—standard neurocritical care, pH, and experimental strategies.

To account for the heterogeneity of study designs, Table 1 provides an overview of all included studies, specifying study type (preclinical vs. clinical), context (acute vs. chronic TBI), population or model, study design, main findings, and relevance to cerebrovascular dysfunction. Given the diversity of methodologies, no quantitative meta-analysis was attempted. Instead, findings were synthesized qualitatively, emphasizing converging mechanisms across models and highlighting discrepancies between preclinical and clinical evidence. This approach enabled us to capture both the translational potential and the limitations of the current evidence base.

## 3. Cerebrovascular Pathophysiology Following TBI

### 3.1. Primary vs. Secondary Vascular Injury

Traumatic brain injury triggers a series of cerebrovascular events that can be categorized into primary and secondary vascular injuries, each playing a distinct role in the pathogenesis of acute and delayed brain dysfunction.

Primary vascular injury occurs at the moment of trauma and results from mechanical forces such as acceleration–deceleration, rotational strain, and direct impact. These forces can lead to immediate disruption of cerebral vessels, including shearing of capillaries and small arterioles, leading to microhaemorrhages, petechial bleeding, and disruption of the neurovascular unit (NVU) [29,30]. Direct injury to major cerebral arterial or venous vessels should be considered, particularly in cases of penetrating trauma. Beyond combat-related injuries, common causes in civilian settings include air gun wounds and dog bites in the pediatric population. In contrast, in adults, there has been a marked increase in firearm-related injuries [20,21]. Arterial laceration will produce local hemorrhagic lesions that extend according to vessel caliber and the velocity of the foreign body, but immediately after, an ischemic cascade will develop in the distribution territory of the damaged artery. Postmortem studies have confirmed the presence of acute intraparenchymal bleeding and endothelial damage shortly after TBI [8]. The microvascular trauma often parallels the distribution of diffuse axonal injury, especially in areas such as the corpus callosum and deep white matter [14].

Additionally, in severe trauma mechanisms like strangulation or neck compression, global cerebral ischemia may occur due to impaired arterial inflow or venous outflow obstruction. Laaksonen et al. reported frequent ischemic changes and brain edema in forensic TBI cases involving asphyxia and blunt trauma to the neck, emphasizing the mechanism-specific nature of primary vascular insults [19]. Secondary vascular injury, on the other hand, develops over hours to days and involves a complex interplay of neuroinflammation, oxidative stress, vascular dysregulation, and BBB breakdown [5,7,8]. These processes develop gradually, often in tissue that initially seemed unaffected by mechanical damage.

The clinical importance of distinguishing between primary and secondary vascular injury lies in the therapeutic window: while primary damage is irreversible, secondary mechanisms can be potentially targeted. Interventions that focus on cerebral perfusion, BBB integrity, or vascular inflammation are key to improving outcomes after TBI [2].

**Key takeaways:** Taken together, the evidence shows that primary vascular lesions are well characterized in histopathological studies, whereas secondary mechanisms are mainly demonstrated in experimental models. Distinguishing between the two is not only conceptual but has direct therapeutic relevance: interventions can only target the delayed, evolving processes. Future research should focus on defining the time-sensitive therapeutic window for secondary vascular injury and on developing strategies to stratify patients at admission using imaging or biomarker profiles to guide individualized treatment.

### 3.2. Hypoxic–Ischemic Damage and Blood–Brain Barrier Disruption

One of the most damaging consequences of TBI is the development of secondary hypoxic–ischemic injury, which occurs when cerebral oxygen delivery is impaired relative to metabolic demand. This imbalance often results from decreased cerebral perfusion pressure (CPP), microvascular dysfunction, or disturbances in cerebral autoregulation, and is a key determinant of neurological deterioration and long-term disability following TBI [10,30].

The blood–brain barrier plays a central role in maintaining cerebral homeostasis, protecting the brain from circulating toxins and regulating the exchange of fluids and solutes. Following TBI, the BBB is frequently compromised due to mechanical stress, inflammatory cytokines, oxidative stress, and endothelial dysfunction [8,9]. Endothelial cells, as gatekeepers of the BBB, are particularly vulnerable: exposure to cytokines such as TNF-α and IL-1β upregulates adhesion molecules (ICAM-1, VCAM-1) and promotes leukocyte infiltration [7]. At the same time, excessive production of reactive oxygen species (ROS) and peroxynitrite damages endothelial tight junctions and glycocalyx, leading to plasma protein extravasation and vasogenic edema [3,5].

Endothelial mediators further influence the vascular response. An imbalance between nitric oxide (NO) and endothelin-1 (ET-1) contributes to dysregulated vasomotor tone: decreased endothelial NO synthase (eNOS) activity impairs vasodilation, while increased ET-1 promotes sustained vasoconstriction and ischemia [14]. Simultaneously, activation of matrix metalloproteinases (MMP-2 and MMP-9) breaks down the basal lamina and tight junction proteins, worsening BBB disruption [6,7]. Pericytes, which are vital partners of endothelial cells, rely on platelet-derived growth factor-β (PDGF-β) signaling for vascular stability; its reduction after TBI destabilizes the pericyte–endothelium communication, intensifying barrier breakdown [7]. Moreover, astrocytic aquaporin-4 (AQP4) is often upregulated in pericontusional areas, increasing water influx and contributing to vasogenic edema [7].

Preclinical studies highlight this endothelial vulnerability. Bhowmick et al. demonstrated that pericyte loss and decreased PDGF-β signaling post-TBI result in downregulation of tight junction proteins and elevated serum levels of CNS injury markers (S100B, NSE) [9]. Similarly, Trofimov et al. showed that endothelial activation promotes microvascular thrombosis, further aggravating local hypoxia [3]. Clinical findings support these mechanisms: Fullerton et al. reported widespread vascular leakage and endothelial disruption in postmortem TBI tissue, with capillary-level damage predominating in children and larger-vessel involvement in adults [8].

Similarly, Laaksonen et al. (2024), in a forensic autopsy study, found frequent hypoxic–ischemic lesions in the hippocampus and cerebral cortex, particularly in cases involving strangulation or severe blunt force trauma, where cerebral oxygenation was likely impaired for prolonged periods [14].

From a diagnostic perspective, circulating biomarkers such as ischemia-modified albumin (IMA) reflect systemic oxidative stress and endothelial injury. Elevated IMA levels significantly correlated (*p* < 0.05) with radiologic signs of ischemia and poor outcome [4].

**Key takeaways:** These findings highlight endothelial dysfunction and BBB breakdown as critical mediators of hypoxic–ischemic injury following TBI. Endothelial imbalance—caused by disrupted NO–ET-1 signaling, MMP activation, loss of PDGF-β–pericyte support, and astrocytic AQP4 upregulation—links vascular leakage with neuroinflammation, edema, and microthrombosis. A major challenge is to identify biomarkers and imaging markers that can distinguish between reversible and irreversible BBB damage, which could enable targeted barrier-stabilizing therapies. Combining BBB protection with strategies to enhance oxygen delivery may provide a dual approach to reduce ischemic damage in TBI.

### 3.3. Microvascular Dysfunction and Capillary Transit Time Heterogeneity

After TBI, cerebral microcirculation is frequently impaired even in regions distant from the primary lesion. Microvascular dysfunction involves both structural alterations (capillary loss, pericyte detachment, endothelial swelling) and functional changes in perfusion dynamics.

A central concept is capillary transit time heterogeneity (CTTH), which describes uneven capillary flow leading to inefficient oxygen extraction despite preserved or even increased cerebral blood flow (CBF). In healthy tissue, capillary perfusion is relatively uniform, ensuring efficient oxygen delivery. TBI disrupts this balance, causing flow shunting—some capillaries are hyperperfused, while others receive little or no blood, reducing net oxygen utilization [10,19,30].

Østergaard et al. modelled CTTH and demonstrated that functional hypoxia can occur in the absence of large-vessel occlusion or visible edema [8]. Bragin et al., using high-resolution imaging in a rat TBI model, reported capillary rarefaction and elevated CTTH in the pericontusional cortex, which correlated with reduced oxygen extraction and neuronal hypoxia [19]. Pericytes play a crucial role: Bhowmick et al. showed that pericyte loss destabilizes microvascular flow and increases BBB leakage, reinforcing the link between cellular injury and CTTH [9].

Clinical studies provide converging evidence. Amyot et al. and Haber et al. used ASL and BOLD MRI in chronic TBI patients and found reduced cerebrovascular reactivity and perfusion heterogeneity in structurally normal white matter, suggesting persistent microvascular dysfunction as a driver of post-concussive symptoms [11,12].

**Key takeaways:** Recent modeling and experimental work demonstrates that CTTH leads to functional hypoxia without large-vessel occlusion, a concept that reframes how ischemia after TBI is understood. Preclinical studies highlight pericyte loss and capillary rarefaction as cellular drivers of microvascular dysfunction—findings not yet systematically integrated into clinical care. In our view, the concept of CTTH highlights the need for a paradigm shift in monitoring: current clinical tools often capture global perfusion but overlook microvascular flow heterogeneity. Future work should focus on developing bedside-compatible techniques to quantify CTTH and to determine its prognostic value in TBI patients.

### 3.4. Cerebrovascular Autoregulatory Failure

Cerebral autoregulation refers to the intrinsic ability of cerebral blood vessels to maintain relatively constant CBF across a range of systemic blood pressures. This mechanism ensures stable oxygen and nutrient delivery despite fluctuations in mean arterial pressure (MAP). In the context of TBI, autoregulation is often impaired, leaving the brain vulnerable to both hypoperfusion and hyperemia, which can exacerbate tissue injury and intracranial hypertension [2,10,30].

Beyond NO and ET-1 signaling (as shown in Section 3.2), inflammatory activation of the endothelium increases adhesion molecules (ICAM-1, VCAM-1) that recruit leukocytes and sustain vasoparalysis. Microvascular thrombosis, driven by this endothelial activation, can further impair perfusion at the capillary level, worsening autoregulatory failure [3].

Clinically, autoregulatory impairment is detected using indices such as the pressure reactivity index (PRx), where a positive correlation between ICP and MAP reflects passive vessel behavior and loss of autoregulation [5]. Advanced imaging studies also demonstrate widespread deficits in cerebrovascular reactivity (CVR). For example, Amyot et al. reported that chronic TBI patients exhibited reduced CVR on ASL and BOLD MRI, even in structurally normal regions, suggesting persistent endothelial-mediated vascular dysfunction [9]. Similarly, Haber et al. found that impaired CVR correlated with cognitive and behavioral symptoms, linking autoregulatory deficits to long-term outcome [12].

Therapeutically, autoregulatory failure is an attractive vascular target. One promising strategy involves modulation of the NO–cGMP pathway. Sildenafil, a selective phosphodiesterase-5 (PDE5) inhibitor, prevents cGMP breakdown and amplifies endothelial NO signaling, thereby restoring vasodilatory capacity in TBI models. Preclinical and early clinical studies suggest that sildenafil can improve CVR and perfusion selectively in regions of endothelial dysfunction, without affecting normal vessels [13,31].

**Key takeaways**: Evidence increasingly shows that autoregulatory failure after TBI is driven by the endothelium and is therefore potentially modifiable. What has been missing from most reviews is an integrated perspective: endothelial molecular signals (NO, ET-1, PDE5) are not just basic science findings but have direct diagnostic links (PRx, CVR imaging) and therapeutic implications (sildenafil and other endothelial-targeted drugs). By framing autoregulation as an endothelial phenotype, we highlight a translational opportunity: patients could be stratified at the bedside based on vascular reactivity profiles and treated with agents designed to restore endothelial signaling. This positions cerebrovascular autoregulation not merely as a prognostic marker but as a therapeutic target within a critical time window.

### 3.5. Post-Traumatic Cerebral Infarction (PTCI)

Although hemorrhagic lesions are the hallmark of traumatic brain injury, ischemic strokes—particularly post-traumatic cerebral infarctions (PTCI)—represent a serious and often underrecognized complication. These infarcts can occur in the acute or subacute phase after TBI and are associated with significantly increased morbidity and mortality [6,30].

The pathophysiology of PTCI is multifactorial. It often results from a combination of global and regional hypoperfusion, brain herniation with vascular compression, vasospasm, microthrombi, and autoregulation failure [14,30]. In many cases, PTCI occurs due to secondary insults, including systemic hypotension, raised ICP, or impaired CPP, rather than direct vascular injury.

In a clinical study of 150 TBI patients, Shah and Langhnoja found that 16% developed PTCI, with most infarcts appearing within the first two weeks post-injury [6]. The middle cerebral artery (MCA) territory was the most affected (47.6%), followed by the anterior and posterior cerebral arteries. Risk factors significantly associated with infarction included low Glasgow Coma Scale (GCS < 8), systolic blood pressure < 90 mmHg, subarachnoid hemorrhage (SAH), and radiological evidence of brain herniation. Mortality in the PTCI group was 80%, compared to 12.5% in patients without infarction, highlighting a statistically significant difference (*p* < 0.001) [6].

Autopsy data further confirm the prevalence of ischemic changes in TBI patients. Laaksonen et al. reported frequent hypoxic–ischemic neuronal damage in forensic TBI cases, particularly in the hippocampus, cortex, and deep gray matter. Notably, infarcts were more prominent in deaths involving mechanical asphyxia or prolonged hypoxia, suggesting that PTCI may evolve silently, especially in patients without continuous neuromonitoring [14].

Mechanical compression of cerebral vessels during herniation is a well-documented cause of infarction. Post-traumatic vasospasm, especially in the context of SAH, can decrease perfusion in downstream territories, mimicking aneurysmal stroke patterns [14]. Inflammatory activation of the endothelium can also promote microvascular thrombosis, contributing to “no-reflow” phenomena and microinfarcts [3]. The role of autoregulatory failure in PTCI is increasingly recognized. When cerebral vessels lose their ability to adjust to changes in MAP, pressure-passive perfusion occurs, making the brain highly vulnerable to systemic hypotension or ICP surges [4,14]. Even minor hemodynamic fluctuations can trigger watershed infarctions or deep territorial ischemia in these patients.

Detection of PTCI often requires multimodal imaging because early CT scans may miss evolving infarcts. Diffusion-weighted MRI (DWI) is the most sensitive modality, while CT perfusion and transcranial Doppler can provide early hemodynamic clues. In patients with equivocal findings or fluctuating neurological status, these tools may be essential in preventing delayed deterioration [17].

From a management perspective, early identification of patients at risk for PTCI is essential. Protocols such as THE MANTLE bundle, which focus on optimizing cerebral oxygenation and perfusion, may help prevent secondary ischemic events in severe TBI [2]. In certain cases, decompressive craniectomy, osmotherapy, or targeted blood pressure augmentation may be needed to restore perfusion and avoid irreversible damage.

**Key takeaways:** PTCI is a severe and often preventable consequence of cerebrovascular compromise in TBI. Risk stratification, use of multimodal imaging techniques, aggressive hemodynamic optimization, and early imaging are essential in reducing its incidence and improving patient outcomes.

### 3.6. Oxidative Stress, Neuroinflammation, and Microthrombi

Oxidative stress and neuroinflammation are closely connected processes that play a key role in the development of secondary brain injury after TBI. Along with endothelial dysfunction and loss of autoregulation, they contribute to the formation of microthrombi, which impair microvascular perfusion and lead to tissue hypoxia and infarction [3,4,14].

Immediately after trauma, resident microglia become activated and release pro-inflammatory cytokines such as IL-1β, TNF-α, and IL-6, which increase vascular permeability and attract neutrophils and monocytes to the injury site [4,17]. The activated inflammatory cells produce reactive oxygen species (ROS) and nitric oxide, amplifying oxidative stress within the neurovascular unit [8].

This redox imbalance leads to damage of endothelial cells, mitochondrial dysfunction in neurons and glia, and disruption of tight junction proteins, further exacerbating vasogenic edema and neuronal injury. Trofimov et al. demonstrated in experimental TBI models that oxidative stress and endothelial activation promote microvascular thrombosis, which correlates with cognitive impairment and persistent microvascular hypoperfusion [3,5].

A key pathophysiological consequence of this cascade is the formation of microthrombi, which occlude capillaries and arterioles, triggering localized ischemia. Histological studies reveal fibrin deposition and platelet aggregation in injured microvessels as early as hours after injury [5]. Due to microvascular obstruction, these microthrombi contribute to the so-called “no-reflow” phenomenon, where reperfusion is inadequate even after ICP normalization.

**Key takeaways:** Overall, the evidence shows that oxidative stress, inflammation, and microvascular thrombosis are key factors in the progression of secondary injury. Addressing these interconnected pathways may provide therapeutic opportunities, especially when combined with strategies that maintain BBB integrity and enhance cerebral perfusion.

**Figure 2:**
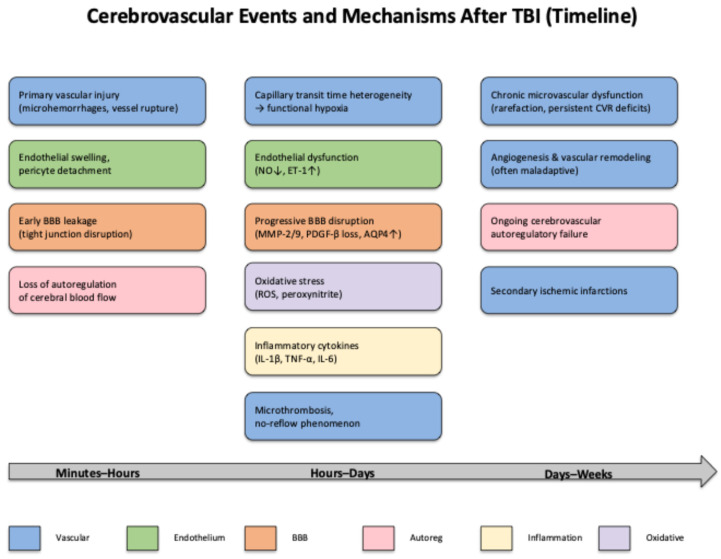
Timeline of cerebrovascular events and mechanisms after TBI.

A summary of key pathophysiological mechanisms, their consequences, and the type of supporting evidence is presented in Table 2. The temporal evolution of these mechanisms is schematically represented in Figure 2, providing an integrated view of how acute vascular damage transitions into delayed cerebrovascular dysfunction.

The diagram illustrates the temporal sequence of cerebrovascular events and mechanisms following TBI, from acute (minutes–hours) to chronic phases (weeks), highlighting primary vascular injury, endothelial and BBB disruption, oxidative stress, neuroinflammation, and microthrombosis, which converge toward impaired autoregulation of cerebral blood flow, secondary ischemic infarctions, and chronic cerebrovascular dysfunction.

A deeper understanding of these pathophysiological mechanisms highlights the importance of diagnostic approaches that can identify cerebrovascular dysfunction early and accurately. The next section examines how advanced imaging, bedside monitoring, and circulating biomarkers can turn mechanistic insights into practical clinical tools.

## 4. Diagnostic Techniques and Biomarkers

Cerebrovascular dysfunction after TBI is not always detectable on standard imaging. A range of advanced methods, including functional imaging, bedside monitoring, and circulating biomarkers, have been developed to identify perfusion issues, barrier disruption, and vascular reactivity.

Functional imaging and physiological monitoring now enable direct assessment of perfusion dynamics, BBB integrity, and cerebrovascular reactivity. Techniques such as perfusion CT, diffusion- and perfusion-weighted MRI, arterial spin labeling, dynamic contrast-enhanced MRI, BOLD MRI with hypercapnia, Xenon CT, and PET offer complementary insights into regional blood flow, oxygen extraction, and barrier permeability.

Meanwhile, bedside monitoring tools—including the pressure reactivity index (PRx), brain tissue oxygenation (PbtO_2_), jugular venous saturation, transcranial Doppler, and near-infrared spectroscopy—provide continuous physiological assessment. These methods detect dynamic changes in autoregulation, oxygen delivery, and perfusion balance, often identifying secondary insults earlier than standard imaging.

Together, these approaches contribute to a more comprehensive picture of secondary vascular injury after TBI. Their main applications, strengths, and limitations are summarized in Table 3.

**Key takeaways:** While conventional CT and MRI identify structural lesions, advanced modalities, such as ASL, DCE-MRI, BOLD-fMRI, and PET, allow for the assessment of perfusion, vascular reactivity, and barrier integrity. A combined structural–functional approach provides a more comprehensive picture of vascular injury progression. From our perspective, the next step is to move beyond descriptive imaging toward prognostic imaging markers that can guide therapeutic decisions in real time. Future studies should focus on validating advanced modalities such as ASL or DCE-MRI against clinical outcomes, and on integrating multimodal imaging into bedside monitoring protocols. In our view, the greatest potential lies in combining perfusion and barrier-permeability imaging with biomarker panels, creating a multimodal diagnostic framework that can stratify patients according to vascular risk and therapeutic responsiveness.

### Biomarkers of Cerebrovascular Integrity, Ischemic Stress, and Inflammation

This section synthesizes circulating and imaging-derived biomarkers that reflect the integrity of the blood–brain barrier and the neurovascular unit, as well as ischemic/oxidative stress, and systemic and central inflammatory responses relevant to post-traumatic brain injury cerebrovascular dysfunction. These circulating indicators may complement imaging and physiologic monitoring by providing early, minimally invasive signals of vascular compromise and potential for risk stratification. Table 4 summarizes current known biomarkers and their clinical relevance in TBI.

- Ischemia-Modified Albumin (IMA): a modified form of human serum albumin that results from oxidative stress and ischemia. It primarily affects the metal-binding domain of the protein. Elevated IMA levels have been shown to reflect early ischemic changes in cerebral tissues, even in the absence of overt infarction on imaging. Radwan et al. demonstrated that IMA concentrations were significantly higher in patients with moderate-to-severe TBI than in healthy controls. These levels correlated with CT findings of ischemia, Glasgow Coma Scale (GCS) scores, and neurological outcome. As such, IMA may serve as a sensitive and accessible biomarker of BBB dysfunction, microvascular occlusion, and oxidative stress [4].
- Neuron-Specific Enolase (NSE): A glycolytic enzyme found in neurons and neuroendocrine cells. Its presence in serum indicates neuronal injury and BBB permeability. In TBI, elevated serum NSE levels have been linked to worse outcomes, longer ICU stays, and higher mortality. Its relatively short half-life (~24 h) allows for sequential monitoring of injury progression and treatment response.
- S100 calcium-binding protein B (S100B), a calcium-binding protein predominantly expressed by astrocytes, is released into the circulation following blood–brain barrier disruption and glial injury. Elevated serum levels of S100B within the first hours after TBI have been correlated with the presence of intracranial lesions (sensitivity > 85%, specificity ~60%; *p* < 0.05) [32], increased risk of poor neurological outcome, and mortality. Due to its sensitivity and early rise, S100B has been incorporated into prognostic scoring systems and is increasingly recognized as a clinically relevant biomarker of cerebrovascular compromise in TBI [9,32].
- Interleukin-6 (IL-6): a proinflammatory cytokine that plays a central role in post-injury immune activation and endothelial inflammation. Elevated IL-6 levels have been linked to poorer neurological recovery, prolonged edema, and increased infarct volume in both experimental and clinical settings [7].
- The neutrophil-to-lymphocyte ratio (NLR) and the platelet-to-lymphocyte ratio (PLR) have emerged as easily obtainable and cost-effective biomarkers of systemic inflammation and vascular stress. High NLR and PLR values on admission have been associated with unfavorable outcomes (*p* < 0.01), likely reflecting a systemic inflammatory state contributing to microvascular thrombosis and BBB disruption [6].

**Key takeaways:** Circulating biomarkers provide valuable insights into BBB integrity, ischemic stress, and inflammatory activation subsequent to TBI. These markers augment imaging and monitoring modalities by delivering minimally invasive indicators of vascular compromise. We assert that the future trajectory of biomarker research resides in the development of combinatorial biomarker panels, rather than dependence on individual molecules. The integration of vascular, neuronal, and inflammatory markers into predictive models may facilitate earlier patient stratification and inform targeted therapeutic interventions.

The next logical step is to turn these diagnostic insights into therapeutic interventions. Section 5 will thus focus on current treatment strategies and experimental approaches aimed at directly targeting the mechanisms of vascular injury discussed above.

## 5. Therapeutic Interventions and Experimental Strategies

Building upon the diagnostic advancements outlined previously, therapeutic research has progressively concentrated on targeting the vascular mechanisms responsible for secondary brain injury. The subsequent section provides a review of both established neurocritical care practices and emerging experimental strategies.

### 5.1. Standard Neurocritical Care

The foundation of TBI management in the acute phase focuses on preserving cerebral perfusion, preventing secondary injury, and ensuring adequate oxygen and substrate delivery to the injured brain. Standard neurocritical care protocols aim to control ICP, maintain CPP, and optimize systemic physiology, especially in patients with impaired autoregulation and vascular compromise.

**Intracranial Pressure and Cerebral Perfusion Management**

Current international guidelines recommend maintaining an ICP below 22 mmHg and a CPP between 60 and 70 mmHg, although these thresholds may vary depending on patient age, autoregulatory status, and injury severity [34]. Elevated ICP impairs cerebral venous outflow, reduces perfusion, and contributes to secondary ischemia. Interventions to control ICP include

- Head elevation and sedation;
- Hyperosmolar therapy (e.g., mannitol, hypertonic saline);
- Controlled ventilation (to prevent hypercapnia-induced vasodilation);
- Decompressive craniectomy in refractory cases.

Monitoring individualized CPP targets based on autoregulatory metrics, such as PRx, has gained traction, allowing clinicians to tailor perfusion goals to real-time cerebral vascular responsiveness [2].

**Multimodal Monitoring and Targeted Protocols**

Advanced neurocritical care integrates multimodal monitoring to detect evolving insults and guide therapy. One of the most structured approaches is the MANTLE protocol, introduced by Godoy et al. [2], which proposes

- Maintaining CPP ≥ 70 mmHg;
- PbtO_2_ ≥ 20 mmHg;
- Avoidance of hypocapnia;
- Temperature control;
- Early identification and correction of autoregulatory dysfunction.

This tiered algorithm enables the early detection of hypoperfusion and hypoxia before irreversible damage occurs, promoting neuroprotective interventions that align with the underlying pathophysiology [2].

**Oxygenation and Ventilation Strategies**

Inadequate cerebral oxygenation is a significant risk factor for secondary ischemic injury. Maintaining normoxia (PaO_2_ > 80 mmHg) and normocapnia (PaCO_2_ around 35–40 mmHg) is crucial. Hyperventilation can temporarily decrease ICP through vasoconstriction, but sustained hypocapnia may cause cerebral hypoperfusion, especially in patients with impaired autoregulation [2]. Therefore, ventilation must be carefully adjusted based on PbtO_2_ and end-tidal CO_2_ monitoring.

**Hemodynamic Optimization and Volume Management**

Maintaining adequate MAP is essential for sustaining CPP, especially when autoregulation is impaired. Norepinephrine is the preferred vasopressor in most cases. Preventing hypotension (SBP < 90 mmHg) is critical, as it is strongly linked to higher mortality in TBI [2]. Monitoring volume status carefully is important to avoid both hypovolemia (which risks ischemia) and hypervolemia (which risks cerebral edema).

**Key takeaways:** The next phase in standard care should focus on dynamic, physiology-based protocols. Instead of relying on fixed ICP and CPP thresholds, clinical management could incorporate real-time cerebrovascular reactivity, oxygenation, and autoregulatory status into continuously updated treatment goals. This approach would enable the development of adaptable treatment protocols, supported by bedside multimodal monitoring and machine learning, to detect early signs of vascular decompensation and facilitate timely interventions. In our opinion, integrating such adaptive strategies into routine neurocritical care could be an effective way to reduce secondary ischemic injury in the upcoming years.

### 5.2. Pharmacological Approaches

Pharmacological management in TBI has traditionally focused on ICP control, sedation, and seizure prophylaxis. However, increasing attention is being given to agents that may influence cerebrovascular function and inflammation. Several pharmacological strategies are currently under investigation or in clinical use for their effects on vascular tone, endothelial integrity, neuroinflammation, and oxidative stress.

**Sedation and Cerebrovascular Modulation**

**Propofol**

Propofol remains the sedative of choice in severe TBI due to its rapid onset, controllable duration, and ICP-lowering properties. It reduces cerebral metabolic rate, suppresses excitotoxicity, and promotes vasoconstriction, decreasing CBF and ICP. However, its impact on cerebral autoregulation is dose-dependent, and excessive use may reduce CPP in hypotensive patients [34].

**Vasopressors and CPP Optimization**

Norepinephrine is the first-line vasopressor for maintaining adequate MAP and CPP. In patients with impaired autoregulation, vasoactive support helps prevent pressure-passive perfusion. Although it is not cerebrovascular selective, maintaining systemic perfusion pressure is essential for ensuring cerebral oxygen delivery in the injured brain [2].

Some evidence supports the targeted use of vasodilators, such as sildenafil, to improve CVR. Kenney et al. demonstrated in a pilot trial that sildenafil enhanced CVR in TBI patients, suggesting a potential role for NO/cGMP pathway modulation in restoring vascular responsiveness [13]. However, recent clinical data indicate that prolonged vasopressor therapy, especially with norepinephrine, may have unintended long-term cerebrovascular effects. A retrospective study of TBI patients who underwent decompressive craniectomy found a statistically significant link between the duration of norepinephrine use and the development of posttraumatic hydrocephalus (PTH) [27]. This implies that sustained vasopressor-induced cerebral pulsatility and changes in venous drainage could disrupt CSF hydrodynamics, leading to secondary complications beyond the acute phase.

**Anti-Inflammatory and Antioxidant Therapies**

Based on the mechanisms outlined in Section 3.6, several pharmacological strategies have been explored to reduce oxidative stress and neuroinflammation after TBI. Instead of targeting neuronal injury directly, these methods aim to stabilize the neurovascular unit through anti-inflammatory, antioxidant, and endothelial-protective effects. Several agents have demonstrated promise in modulating these pathways.

- Steroids, though historically used, have been shown to worsen outcomes in the CRASH trial and are now contraindicated in routine TBI management.
- Melatonin and minocycline have shown neurovascular protection in preclinical models through anti-inflammatory and anti-apoptotic mechanisms; however, data in humans remain limited. Aside from its anti-inflammatory effects, minocycline inhibits microglial activation, decreases iNOS expression and nitric oxide release, downregulates IL-6 and TNF-α, and suppresses MMP-9 activity, thereby maintaining tight junction proteins and BBB integrity [35].

**Experimental Vasoactive Compounds**

*Tanshinone IIA*

Su et al. investigated Tanshinone IIA, a compound derived from *Salvia miltiorrhiza*, which exhibits antioxidant and anti-inflammatory properties. In a murine TBI model, Tanshinone IIA reduced infarct size, suppressed ROS production, and preserved vascular architecture through upregulation of the miR-124-5p/FoxO1 axis [7]. This suggests a multimodal vasoprotective mechanism relevant to early microvascular preservation.

**Antithrombotic and Endothelial-Stabilizing Agents**

Considering the role of microthrombi in ischemia related to TBI, antithrombotic treatments such as low-dose heparin and antiplatelet therapy have been suggested in controlled studies. Nevertheless, the danger of bleeding presents a major obstacle. Current research focuses on creating treatments that support endothelial stability and inhibit the activation of the coagulation process without raising hemorrhage risk.

**Key takeaways:** Pharmacological approaches targeting cerebrovascular dysfunction in TBI have been promising in preclinical studies. However, clinical evidence remains limited to a few small pilot or exploratory trials, and the effectiveness and dosing of these treatments have not been validated in larger populations. Progress in pharmacological strategies will likely depend on shifting from single-agent treatments to time-sensitive combination therapies that are tailored to the different phases of vascular injury. For example, using antioxidants and BBB stabilizers early on might reduce acute endothelial damage, while anti-inflammatory drugs and vasomodulators could be more beneficial during the subacute stage.

### 5.3. Experimental Therapies

Because conventional therapies have limited success in treating microvascular dysfunction and secondary ischemic injury, new experimental strategies have been developed to target specific parts of the neurovascular unit. These approaches aim to maintain endothelial integrity, regulate inflammatory signaling, and encourage angiogenesis and vascular repair after TBI.

**MicroRNA and Gene-Modulating Therapies**

One of the most promising molecular pathways involves microRNAs (miRNAs), which regulate gene expression related to endothelial stability, oxidative stress, and apoptosis.

Similar strategies involving siRNA or CRISPR-based modulation of vascular and inflammatory targets are being researched, although none have yet been applied clinically in TBI.

**Cell-Based Therapies and Angiogenesis**

Cell-based therapies represent another frontier in neurovascular regeneration. Research involving mesenchymal stem cells (MSCs) and endothelial progenitor cells (EPCs) has demonstrated potential to

- Enhance angiogenesis;
- Secret neurotrophic and anti-inflammatory factors;
- Stabilize the BBB.

These effects may restore microvascular perfusion and accelerate structural and functional recovery [30]; however, concerns about cell delivery, homing, immunogenicity, and long-term safety limit current clinical translation.

**Exosomes and Extracellular Vesicles (EVs)**

Exosomes are nano-sized extracellular vesicles released by various cell types, including stem and endothelial progenitor cells, and have emerged as a promising acellular therapeutic strategy in TBI. Their ability to cross the BBB with low immunogenicity makes them attractive for the targeted delivery of therapeutic cargo.

These vesicles transport microRNAs, proteins, and lipids that modulate inflammation, promote angiogenesis, and support neuroregeneration. In a key study, Gao et al. (2018) demonstrated that exosomes derived from endothelial colony-forming cells could restore BBB integrity, increase microvascular density, and enhance expression of tight junction proteins in a murine model of TBI [15].

Further support is provided by Wang et al. (2022), who reviewed the therapeutic role of cell-derived exosomes in central nervous system injuries. They reported that exosomes could deliver regulatory molecules that reduce neuroinflammation, suppress oxidative stress, and stimulate angiogenesis, potentially replacing or enhancing the effects of direct cell transplantation [16].

These findings indicate that exosomes might address several limitations of cell-based therapy, such as cell survival, delivery, and immune compatibility concerns, while maintaining strong regenerative effects on the neurovascular unit.

**BBB Stabilizers and Tight Junction Modulators**

Molecules like angiopoietin-1, VEGF, and MMP inhibitors are being tested to prevent capillary leak and tight junction breakdown [28]. While some have demonstrated effectiveness in animal models, human trials are still in early stages or have not yet been conducted.

**Antioxidants and Free Radical Scavengers**

Beyond conventional agents like vitamin E or N-acetylcysteine, newer antioxidant compounds targeting mitochondrial dysfunction and ROS production (e.g., MitoQ, Edaravone) have shown promise in preserving vascular reactivity and capillary perfusion [25]. Their ability to reduce microthrombus formation and inflammation may benefit both the acute and chronic phases of TBI.

**Perivascular Macrophages (PVMs)**

In models of cerebral hemorrhage, bexarotene administration increased the expression of phagocytosis receptors such as CD36 and Axl in macrophages, thereby reducing hematoma volume and enhancing neurological recovery. Future studies will be able to determine whether metabolic manipulation of PVMs can stimulate their protective effects. The possibility of transplanting PVMs at specific times, at the level of the lesion, to facilitate neurorecovery is also of interest [25].

**Key takeaways:** Emerging experimental strategies, including exosome therapy, stem cell transplantation, and microRNA modulation, target the neurovascular unit and have demonstrated substantial mechanistic benefits in animal models. Representative examples and supporting evidence are summarized in Table 5. To date, however, no clinical trials have validated these therapies in the context of TBI.

In our view, combining exosome-derived biomolecules with pharmacological agents such as vasomodulators could provide synergistic protection of the microvasculature. Moreover, the development of standardized protocols for cell- and exosome-based therapies, including optimal dosing and delivery routes, is urgently needed to ensure the clinical translation of these therapies. We also believe that future studies should prioritize comparative evaluations across models, enabling a clearer understanding of which experimental strategies hold the most realistic potential for bedside application.

## 6. Future Directions

While significant advances have been made in understanding the vascular pathology of TBI, future directions must focus on translating these insights into targeted interventions. Three key mechanisms have emerged as viable therapeutic targets for limiting the cascade of posttraumatic ischemic injury:

1. **Secondary BBB Disruption**

Secondary BBB represents a potentially modifiable event, and targeting this phase with agents that stabilize endothelial function or inhibit matrix metalloproteinases (MMPs) may reduce vasogenic edema, neuroinflammation, and secondary cell death. Future therapies might include pharmacological agents such as angiopoietin-1 mimetics and selective COX-2 inhibitors, designed to strengthen and maintain BBB integrity during the subacute phase.

2. **Oxidative Stress, Neuroinflammation, and Microthrombosis**

Anti-inflammatory and antioxidant therapies remain among the most promising translational strategies for TBI. Recent studies show a convergence of evidence supporting selective COX-2 inhibitors (e.g., celecoxib) and minocycline as consistent modulators of vascular inflammation and BBB disruption.

The next step is to validate these agents in well-designed clinical trials, ideally combined with vascular stabilizers or antioxidants, to enhance neurovascular protection. Table 6 summarizes key pharmacological agents tested in preclinical or clinical studies, highlighting their mechanisms of action, cerebrovascular effects, and the level of supporting evidence [29,30,35,36].

3. **Cerebrovascular Autoregulatory Failure**

Among vascular targets, autoregulatory impairment remains one of the most clinically relevant yet under-addressed phenomena in TBI. Building on the early evidence reviewed in Section 5.2, sildenafil has emerged as a promising vasomodulator capable of restoring cerebrovascular reactivity. Their use in experimental protocols targeting cerebrovascular reactivity is highlighted in Table 7 [18,28,31].

Sildenafil seems to be a promising adjunct for enhancing cerebrovascular function and outcomes in TBI, stroke, and hypoxic brain injury. Its mechanism—boosting NO–cGMP signaling—improves cerebral perfusion and offers downstream neuroprotective effects [31]. Optimizing dosing and timing will be essential for integrating sildenafil as a neurovascular therapy.

**Toward Multimodal Therapeutic Combinations**

A combination of therapies that act synergistically on different aspects of vascular dysfunction may offer the most effective neuroprotection. Table 8 outlines a hypothetical therapeutic protocol incorporating agents with complementary actions that remains to be validated in clinical trials.

## 7. Conclusions

Since Ancient Egypt, when the Edwin Smith Papyrus first documented the elevation of the head in severe brain trauma [37], until today’s molecular understanding of pathophysiological processes, the prognosis of severe traumatic brain injury remains a global challenge. Secondary cerebrovascular processes play a crucial role in patient outcomes. Although previous research has examined these pathways, this synthesis offers new insights that could transform monitoring methods and treatment strategies. It may be time to shift the therapeutic perspective for these cases, based on the latest research in a field where the cascade of adverse events can be slowed, stopped, or even reversed.

First, we emphasize the need to move beyond simple descriptions of vascular pathology toward a comprehensive, multimodal diagnostic approach. In our view, combining perfusion and permeability imaging with biomarker panels that can distinguish reversible from irreversible blood–brain barrier disruption is a crucial step for patient stratification and targeted treatment. This strategy could change the BBB from just a static damage marker into a dynamic, adjustable therapeutic target.

Second, by emphasizing capillary transit time heterogeneity as a determinant of functional hypoxia, we propose a paradigm shift in monitoring. Current bedside tools capture global perfusion but overlook microvascular heterogeneity; future research must develop clinically compatible methods to quantify CTTH and validate its prognostic role in TBI.

Third, we reframe cerebrovascular autoregulation as an endothelial phenotype rather than a static physiological parameter. This conceptual shift enables the stratification of patients based on vascular reactivity profiles and allows for tailored therapies—such as PDE-5 inhibitors—to restore endothelial signaling within a critical therapeutic window.

Building on these mechanistic insights, we further promote the idea that progress depends on time-sensitive, multimodal treatment combinations rather than individual agents. As outlined in our proposed therapeutic framework, synergistic protocols may include early BBB stabilizers (e.g., angiopoietin-1 mimetics), antioxidants such as Tanshinone IIA or mitochondrial-targeted compounds, selective anti-inflammatory drugs (minocycline, celecoxib), vasomodulators restoring autoregulation (sildenafil), and adjunctive strategies addressing endocrine dysfunction and microthrombosis. Although hypothetical, such integrated regimens represent a realistic path forward and highlight the opportunity to match interventions with the evolving phases of vascular injury.

In conclusion, the novelty of this revision lies in presenting a unified vascular perspective that links biomarkers, imaging, autoregulatory profiling, and multimodal therapies into a single continuum. The most crucial next steps for research are (i) validating prognostic imaging and biomarker signatures that guide therapy in real time, (ii) testing adaptive neurocritical care protocols supported by bedside monitoring and machine learning, and (iii) evaluating multimodal therapeutic strategies through rigorously designed clinical trials. We believe that adopting this comprehensive approach could finally enable the field to reduce secondary ischemic injury and achieve more significant recovery after TBI.

## Figures and Tables

**Table 1:** Summary of included studies.

First Author, Year	Study Type	Context	Population/Model	Design	Main Findings	Relevance to Cerebrovascular Dysfunction
**Fullerton et al., 2024** [8]	Clinical	Acute TBI	Pediatric and adult patients	Observational study	Age-specific BBB disruption patterns	Supports BBB vulnerability as central mechanism
**Bhowmick et al., 2019** [9]	Preclinical	Acute TBI	Mouse (controlled cortical impact)	Experimental	Pericyte loss → BBB permeability ↑, S100B/NSE ↑	Pericyte dysfunction drives BBB failure
**Østergaard et al., 2014** [10]	Clinical + Modeling	Acute & chronic	Perfusion imaging and modeling	Theoretical framework	Introduced CTTH; functional hypoxia despite normal CBF	Links microvascular heterogeneity to ischemia
**Amyot et al., 2018** [11]	Clinical	Chronic TBI	Human patients	Imaging study (ASL MRI)	Persistent hypoperfusion and impaired CVR	Demonstrates chronic vascular dysfunction
**Haber, 2018** [12]	Clinical	Chronic TBI	Human patients	Imaging study (CVR mapping)	Reduced CVR in normal-appearing white matter	Suggests long-term autoregulatory impairment
**Kenney et al., 2018** [13]	Clinical	Chronic TBI	Human patients	Pilot interventional trial	Sildenafil improved CVR	NO/cGMP modulation restores autoregulation
**Shah, 2024** [6]	Clinical	Acute TBI	Human patients	Observational study	16% developed PTCI, MCA most affected	Confirms PTCI as major complication
**Laaksonen, 2024** [14]	Clinical	Acute TBI	Forensic autopsy cases	Postmortem study	Frequent ischemic lesions (hippocampus, cortex, deep gray)	Highlights underrecognized ischemic burden
**Radwan, 2021** [4]	Clinical	Acute TBI	Human patients	Observational study	Elevated IMA correlated with severity and poor outcome	Validates IMA as a biomarker of ischemia
**Su, 2024** [7]	Preclinical	Acute TBI	Murine model	Experimental	Tanshinone IIA ↓ infarct size and oxidative stress	Promising vasoprotective agent
**Gao, 2018** [15]	Preclinical	Acute TBI	Mouse (exosomes)	Experimental	Exosomes restored BBB, ↑ microvascular density	Supports exosome therapy for vascular repair
**Wang, 2022** [16]	Preclinical	CNS injury	Rodent models	Narrative review	Exosomes ↓ inflammation, ↑ angiogenesis	Highlights translational potential of EVs
**Honda, 2016** [17]	Clinical	Acute TBI	Severe TBI patients	Imaging study	Quantified early cerebral circulation disturbance	Supports perfusion imaging for ischemia detection
**Kilicarslan, 2019** [18]	Preclinical	Acute TBI	Rat head trauma model	Experimental	Sildenafil reduced inflammation and improved outcomes	Preclinical support for PDE-5 inhibition
**Bragin, 2024** [19]	Preclinical	Acute TBI	Rat model	Experimental	MAGL inhibition attenuated early ischemia	Targets microvascular perfusion and inflammation
**Dobrzeniecki, 2021** [5]	Clinical	Acute TBI	Severe TBI patients	Observational study	Secondary ischemia relates to CVR impairment > ICP	Highlights primacy of CVR failure
**Wijaya, 2019** [20]	Clinical	Acute TBI	Pediatric penetrating TBI (case)	Case report	Bihemispheric injury after air-gun wound	Illustrates primary vascular trauma
**Chen, 2024** [21]	Clinical	Acute TBI	Patients with intracranial gunshot wounds	Retrospective series	Patient factors associated with outcomes	Context for primary vascular injuries
**Wen, 2024** [22]	Preclinical	CNS injury	Murine model	Experimental	Perivascular macrophages contribute to vascular integrity disruption	Highlights immunovascular targets
**Rostami, 2014** [23]	Clinical	Acute TBI	Severe TBI patients	Observational imaging	CBF imaging in NICU contexts	Practical role of perfusion modalities
**Serban, 2024** [24]	Clinical	Post-acute TBI	Patients with decompressive craniectomy	Retrospective cohort	Longer norepinephrine linked with posttraumatic hydrocephalus	Links vasopressors to CSF/vascular dynamics
**Salehi, 2017** [25]	Preclinical/Review	CNS injury	Animal models	Review	Survey of vascular responses and targets	Integrates vascular pathophysiology
**Begemann, 2020** [26]	Clinical/Meta-analysis	TBI	Human studies	Meta-analysis	Anti-inflammatory drugs showed signals of benefit	Supports anti-inflammatory strategies
**Hiskens, 2024** [27]	Preclinical/Review	CNS injury	Rodent models	Review	Selective COX-2 inhibitors showed neuroprotective effects	COX-2 inhibition and BBB preservation
**Kalyani, 2023** [28]	Clinical/Review	Chronic TBI	Human patients	Review	Summarized safety/efficacy of PDE-5 inhibitors	Framework for clinical translation
**Trofimov, 2021** [3]	Clinical	Acute TBI	Human patients	Biomarker study	Microcirculatory markers reflect secondary ischemia	Links endothelial activation to microthrombosis

**Table 2:** Pathophysiological mechanisms in TBI-related cerebrovascular dysfunction.

Mechanism	Process Description	Consequences	Evidence Type	Key References
Primary vascular injury	Mechanical disruption of cerebral vessels during trauma	Microhemorrhages, DAI, petechial bleeding	Autopsy, animal models	Fullerton et al., 2024; Laaksonen et al., 2024; Hausburg et al., 2019 [8,14,32]
BBB disruption	Pericyte loss, endothelial dysfunction, tight junction degradation	Vasogenic edema, neuroinflammation	Animal models, histology	Bhowmick et al., 2019; Fullerton et al., 2024 [8,9]
Microvascular thrombosis	Activation of coagulation, leukocyte adhesion, oxidative stress	Capillary occlusion, hypoxia, inflammation	Animal and postmortem studies	Dobrzeniecki et al., 2021; Hausburg et al., 2019 [5,32]
CTTH (capillary transit time heterogeneity)	Uneven microvascular flow despite normal perfusion	Functional hypoxia, poor oxygen extraction	Theoretical model, neuroimaging	Østergaard et al., 2014 [10]
Autoregulatory failure	Impaired CVR and pressure reactivity (PRx)	Ischemia in hypo/hypertension, secondary infarction	Clinical monitoring studies	Kenney et al., 2018; Amyot et al., 2017 [11,13]

**Table 3:** Comparative imaging modalities for vascular dysfunction in TBI.

Imaging Modality	Target Parameter	Strengths	Limitations	Key References
CT perfusion	CBF, CBV, MTT	Widely available, rapid, detects ischemia	Radiation, contrast agent risk	Honda et al., 2016; Shah et al., 2024 [6,17]
DWI MRI	Cytotoxic edema, infarcts	High sensitivity for acute ischemia	No direct perfusion info, motion artifacts	Shah et al., 2024 [6]
ASL MRI	Non-invasive CBF quantification	No contrast, repeatable	Low SNR, long scan time	Amyot et al., 2017 [11]
DCE-MRI	BBB permeability	Quantitative barrier assessment	Contrast needed, complex post-processing	Fullerton et al., 2024 [8]
BOLD fMRI + CO_2_	CVR assessment	Functional evaluation of autoregulation	Sensitive to motion, not widely available	Haber et al., 2018 [12]
Xenon-CT	Absolute CBF	Bedside-capable, accurate	Limited availability	Østergaard et al., 2014 [10]
PET	Metabolism, oxygen extraction	Flow-metabolism mismatch evaluation	Expensive, limited access	Østergaard et al., 2014 [10]

**Table 4:** Biomarkers of vascular dysfunction and prognosis in TBI.

Biomarker	Biological Role	Clinical Relevance	Timing	Sensitivity/Specificity	Key References	Level of Evidence	Limitations
**IMA**	Oxidative stress marker	Correlates with infarction, severity	Within 6–24 h post-injury	Sensitivity ~80%, specificity ~75% (reported in small cohorts)	Radwan et al., 2020 [4]	Clinical observational cohort	Small sample size (n = 60); single-center; no multicenter validation
**NSE**	Neuronal injury marker	Associated with ICU stay, mortality	Peaks around 24–72 h	Sensitivity ~70%, specificity ~65% (variable across studies)	Bhowmick et al., 2019 [9]	Preclinical + small clinical cohorts	Heterogeneous cut-offs; not standardized across centers
**S100B**	Astrocytic protein, BBB leakage indicator	Used in prognostic scoring	Early, up to 6 h post-injury	Sensitivity > 85% for intracranial lesions; specificity ~60%	Hausburg et al., 2019 [32]	Strong preclinical + validated in multiple small clinical cohorts	High sensitivity but limited specificity; extracranial sources may confound
**IL-6**	Pro-inflammatory cytokine	Linked with edema, worse outcome	Elevated in first 48 h	Sensitivity/specificity values inconsistent	Su et al., 2024 [7]	Observational clinical cohorts	High variability; influenced by systemic inflammation
**NLR/PLR**	Systemic inflammation indices	Predict neurologic outcome	At admission, serial measurements	Sensitivity ~70%, specificity ~68% (cut-offs vary)	Paracino et al., 2024 [33]	Retrospective observational human studies	Cut-offs inconsistent; influenced by comorbidities and systemic infection

Abbreviations: IMA = ischemia-modified albumin; NSE = neuron-specific enolase; S100B = S100 calcium-binding protein B; IL-6 = interleukin-6; NLR = neutrophil-to-lymphocyte ratio; PLR = platelet-to-lymphocyte ratio.

**Table 5:** Representative therapeutic strategies targeting cerebrovascular dysfunction in TBI, selected based on preclinical and clinical evidence.

Therapy Category	Intervention	Mechanism/Target	Evidence Type	Key References
Standard neurocritical care	ICP/CPP optimization, sedation, osmotherapy	Stabilizes perfusion and oxygen delivery	Clinical guidelines	Godoy et al., 2023 [2]
Multimodal protocol	THE MANTLE bundle	Physiological coherence: PRx, CPPopt, PbtO_2_	Prospective clinical model	Godoy et al., 2023 [2]
Pharmacologic agents	Sildenafil, Tanshinone IIA, corticosteroids	Enhance vasodilation, reduce inflammation, support endocrine function	Animal models, pilot trials	Su et al., 2024; Kenney et al., 2018 [7,13]
Antioxidants	MitoQ, Edaravone	Scavenge ROS, protect mitochondria, preserve BBB	Preclinical studies	Salehi et al., 2017; Su et al., 2024 [7,25]
Experimental therapies	Exosomes, miRNA, stem cells	Modulate gene expression, promote angiogenesis, reduce inflammation	Animal studies, preclinical models	Hausburg et al., 2019; Gao et al., 2018 [15,32]

**Table 6:** Anti-inflammatory drugs with cerebrovascular impact in TBI4.

Drug	Mechanism of Action	Cerebrovascular Effects	Dosage (Where Reported)	Evidence Level	Limitations	Key References
**Celecoxib (COX-2 inhibitor)**	Inhibits COX-2 → ↓ Prostaglandins, TNF-α, IL-1β	Preserves BBB, reduces edema and oxidative stress	Typical oral human dose: 200–400 mg/day; animal models variable	Animal models, limited clinical data	No RCTs in TBI; dosing extrapolated from non-TBI use	Hiskens et al., Selective COX-2 Inhibitors, 2024 [27]
**Minocycline**	Reduces microglial activation, ↓ NO, IL-6, TNF-α	Prevents apoptosis, BBB disruption, promotes recovery	Rodents: 45 mg/kg; exploratory human oral dosing (100–200 mg/day)	Strong preclinical data	Clinical evidence minimal; heterogeneous dosing	Bergold et al., Treatment of TBI with Anti-inflammatory Drugs, 2016 [35]
**Progesterone**	Modulates inflammation and promotes remyelination	Reduces edema and neuroinflammation	Human RCTs: 12–16 mg IV q12h	Meta-analysis of RCTs	Mixed efficacy; large trials negative for mortality benefit	Begemann et al., Drugs with Anti-inflammatory Effects, 2020 [26]
**Ibuprofen**	Non-selective COX inhibitor	Weak cerebrovascular protection	Typical human dose: 400–800 mg every 6–8 h	Inconsistent data	Lacks TBI-specific controlled trials; confounded by systemic use	Bergold et al., Treatment of TBI with Anti-inflammatory Drugs, 2024 [35]
**Methylprednisolone**	Corticosteroid, general anti-inflammatory	No benefit, associated with higher mortality	CRASH trial: 30 mg/kg IV bolus + 5.4 mg/kg/h infusion (24–48 h)	CRASH trial, clinical studies	Demonstrated harm in TBI; contraindicated	Kalra et al., Pathogenesis and Management of TBI, 2022 [36]

**Table 7:** Sildenafil dosage strategies and side effects [28].

Study Context	Dosing Strategy	Observed Effects	Side Effects
**Severe TBI (rats)**	10 mg/kg, intraperitoneal, single dose post-injury	Reduced neuronal death within hours	No specific adverse effects reported in rats
**Focal cortical cryoinjury (rats)**	Repeated 10 mg/kg over days	Reduced inflammation and enhanced angiogenesis	No reported systemic side effects
**Ischemic stroke (rats)**	5–10 mg/kg/day, once daily for 1 week post-stroke	Improved perfusion, neurogenesis, and recovery	Well-tolerated in animals, dose-dependent efficacy
**Neonatal hypoxia–ischemia (rats)**	5–10 mg/kg, single or multiple doses; higher dose needed for effect	Reduced apoptosis, preserved hippocampal neurons	Mild transient hypotension at higher doses in neonates
**Chronic TBI (humans, Phase IIa trial)**	50 mg single dose (acute); then 25 mg BID for 8 weeks	Improved CVR; trend toward symptom improvement	No increase in headache; well tolerated overall
**Pulmonary hypertension (humans)**	20 mg TID (standard); up to 80 mg TID tested	Safe in chronic use; basis for neuro trials	Mild flushing, nasal congestion, rarely vision changes
**TBI (humans, high-dose study)**	Titrated up to 80 mg BID (160 mg/day)	Well-tolerated; some minor side effects	Minor effects (epistaxis, diarrhea); no serious events

**Table 8:** Experimental multimodal neurovascular protocol for secondary brain injury in TBI.

Target Mechanism	Therapeutic Agent	Proposed Dose/Regimen	Timing of Administration	Supporting Evidence
BBB stabilization	Angiopoietin-1	Low-dose IV bolus	Within first 6 h post-injury	Su et al., 2024; Salehi et al., 2017 [7,25]
Oxidative stress and mitochondria	Tanshinone IIA/MitoQ	5–10 mg/kg/day (rodents)	First 24–72 h post-TBI	Su et al., 2024 [7]
Neuroinflammation	Minocycline/celecoxib	22.5 mg/kg BID/10–30 mg/kg	First 12–24 h, 3–5 days	Bhowmick et al., 2019; COX-2 Review [9]
Autoregulatory failure	Sildenafil	25–80 mg/day PO or IV	Subacute phase, 24 h–2 weeks	Kenney et al., 2018 [13]
Endocrine dysfunction	Hydrocortisone	50–100 mg/day IV	If low cortisol suspected	Godoy et al., 2023 [2]

## Data Availability

No new data were created in this study. Data sharing is not applicable to this article.

## References

[1] Zhong H., Feng Y., Shen J., Rao T., Dai H., Zhong W., Zhao G. (2025). Global Burden of Traumatic Brain Injury in 204 Countries and Territories From 1990 to 2021. Am. J. Prev. Med..

[2] Godoy D.A., Murillo-Cabezas F., Suarez J.I., Badenes R., Pelosi P., Robba C. (2023). “THE MANTLE” Bundle for Minimizing Cerebral Hypoxia in Severe Traumatic Brain Injury. Crit. Care.

[3] Trofimov A., Dubrovin A., Martynov D., Agarkova D., Trofimova K., Zorkova A., Bragin D.E. (2021). Microcirculatory Biomarkers of Secondary Cerebral Ischemia in Traumatic Brain Injury. Acta Neurochirurgica, Supplementum.

[4] Radwan T.A.M., Fahmy R.S., El Emady M.F.M., Khedr A.S.E.D.M., Osman S.H., ElSonbaty M.I., El-Kholy B.M.B., Thabit M.A., Elkatatny A.M. (2021). Ischemia-Modified Albumin as a Biomarker for Prediction of Poor Outcome in Patients with Traumatic Brain Injury: An Observational Cohort Study. J. Neurosurg. Anesthesiol..

[5] Dobrzeniecki M., Trofimov A., Martynov D., Agarkova D., Trofimova K., Semenova Z.B., Bragin D.E. (2021). Secondary Cerebral Ischemia at Traumatic Brain Injury Is More Closely Related to Cerebrovascular Reactivity Impairment than to Intracranial Hypertension. Acta Neurochirurgica, Supplementum.

[6] Shah A.S., Langhnoja N. (2024). Identifying Clinical and Demographic Predictors of Post-Traumatic Cerebral Infarction in Patients with Traumatic Brain Injury. SSR Inst. Int. J. Life Sci..

[7] Su W., Lv M., Wang D., He Y., Han H., Zhang Y., Zhang X., Lv S., Yao L. (2024). Tanshinone IIA Alleviates Traumatic Brain Injury by Reducing IschemiaReperfusion via the MiR-124-5p/FoxO1 Axis. Mediat. Inflamm..

[8] Fullerton J.L., Hay J., Bryant-Craig C., Atkinson J., Smith D.H., Stewart W. (2024). Pediatric Traumatic Brain Injury and Microvascular Blood-Brain Barrier Pathology. JAMA Netw. Open.

[9] Bhowmick S., D’Mello V., Caruso D., Wallerstein A., Abdul Muneer P.M. (2019). Impairment of Pericyte-Endothelium Crosstalk Leads to Blood-Brain Barrier Dysfunction Following Traumatic Brain Injury. Exp. Neurol..

[10] Østergaard L., Engedal T.S., Aamand R., Mikkelsen R., Iversen N.K., Anzabi M., Næss-Schmidt E.T., Drasbek K.R., Bay V., Blicher J.U. (2014). Capillary Transit Time Heterogeneity and Flow-Metabolism Coupling after Traumatic Brain Injury. J. Cereb. Blood Flow. Metab..

[11] Amyot F., Kenney K., Moore C., Haber M., Turtzo L.C., Shenouda C., Silverman E., Gong Y., Qu B.-X., Harburg L. (2018). Imaging of Cerebrovascular Function in Chronic Traumatic Brain Injury. J. Neurotrauma.

[12] Haber M., Amyot F., Kenney K., Meredith-Duliba T., Moore C., Silverman E., Podell J., Chou Y.-Y., Pham D.L., Butman J. (2018). Vascular Abnormalities within Normal Appearing Tissue in Chronic Traumatic Brain Injury. J. Neurotrauma.

[13] Kenney K., Amyot F., Moore C., Haber M., Turtzo L.C., Shenouda C., Silverman E., Gong Y., Qu B.X., Harburg L. (2018). Phosphodiesterase-5 Inhibition Potentiates Cerebrovascular Reactivity in Chronic Traumatic Brain Injury. Ann. Clin. Transl. Neurol..

[14] Laaksonen J., Mäkinen H., Oura P. (2024). Prevalence of Secondary Brain Injuries and Association with Trauma Circumstances in Neuropathologically Examined Medico-Legal Autopsy Cases with Primary Head Trauma. Leg. Med..

[15] Gao W., Li F., Liu L., Xu X., Zhang B., Wu Y., Yin D., Zhou S., Sun D., Huang Y. (2018). Endothelial Colony-Forming Cell-Derived Exosomes Restore Blood-Brain Barrier Continuity in Mice Subjected to Traumatic Brain Injury. Exp. Neurol..

[16] Wang J., Wang J., Li X., Shu K. (2022). Cell-Derived Exosomes as Therapeutic Strategies and Exosome-Derived MicroRNAs as Biomarkers for Traumatic Brain Injury. J. Clin. Med..

[17] Honda M., Ichibayashi R., Yokomuro H., Yoshihara K., Masuda H., Haga D., Seiki Y., Kudoh C., Kishi T. (2016). Early Cerebral Circulation Disturbance in Patients Suffering from Severe Traumatic Brain Injury (TBI): A Xenon CT and Perfusion CT Study. Neurol. Med. Chir..

[18] Kilicarslan B., Kilicarslan E., Kizmazoglu C., Aydin H.E., Kaya I., Danyeli A.E., Karabekir H.S. (2019). Evaluation of the Efficacy of Sildenafil Citrate Following Severe Head Trauma in an Experimental Rat Model. Turk. Neurosurg..

[19] Bragin D.E., Bragina O.A., Covey D.P., Trofimov A.O., Nemoto E.M., Mayer A.R. (2024). Monoacylglycerol Lipase Inhibition Using ABX-1431 Attenuates Cerebral Ischaemia Early After Traumatic Brain Injury. International Society on Oxygen Transport to Tissue.

[20] Wijaya A.T., Ayusta I.M.D., Niryana I.W. (2019). Air Gun Wound: Bihemispheric Penetrating Brain Injury in a Paediatric Patient. BJR|Case Rep..

[21] Chen Y.-R., Johnson E., Ugiliweneza B., Kim L.H., Shpanskaya K., Boakye M., Tse V. (2024). Intracranial Gunshot Wounds: An Assessment of Patient Characteristics on Surgical Outcomes. Cureus.

[22] Wen W., Cheng J., Tang Y. (2024). Brain Perivascular Macrophages: Current Understanding and Future Prospects. Brain.

[23] Rostami E., Engquist H., Enblad P. (2014). Imaging of Cerebral Blood Flow in Patients with Severe Traumatic Brain Injury in the Neurointensive Care. Front. Neurol..

[24] Șerban N.-L., Florian I.S., Florian I.A., Atena Zaha A., Ionescu D. (2024). Posttraumatic Hydrocephalus as a Complication of Decompressive Craniectomy–Same Old Story, New Perspectives. Front. Surg..

[25] Salehi A., Zhang J.H., Obenaus A. (2017). Response of the Cerebral Vasculature Following Traumatic Brain Injury. J. Cereb. Blood Flow. Metab..

[26] Begemann M., Leon M., van der Horn H.J., van der Naalt J., Sommer I. (2020). Drugs with Anti-Inflammatory Effects to Improve Outcome of Traumatic Brain Injury: A Meta-Analysis. Sci. Rep..

[27] Hiskens M.I., Schneiders A.G., Fenning A.S. (2024). Selective COX-2 Inhibitors as Neuroprotective Agents in Traumatic Brain Injury. Biomedicines.

[28] Kalyani P., Lippa S.M., Werner J.K., Amyot F., Moore C.B., Kenney K., Diaz-Arrastia R. (2023). Phosphodiesterase-5 (PDE-5) Inhibitors as Therapy for Cerebrovascular Dysfunction in Chronic Traumatic Brain Injury. Neurotherapeutics.

[29] Logsdon A.F., Lucke-Wold B.P., Turner R.C., Huber J.D., Rosen C.L., Simpkins J.W. (2015). Role of Microvascular Disruption in Brain Damage from Traumatic Brain Injury. Comprehensive Physiology.

[30] Sandsmark D.K., Bashir A., Wellington C.L., Diaz-Arrastia R. (2019). Cerebral Microvascular Injury: A Potentially Treatable Endophenotype of Traumatic Brain Injury-Induced Neurodegeneration. Neuron.

[31] Xiong Y., Wintermark P. (2022). The Role of Sildenafil in Treating Brain Injuries in Adults and Neonates. Front. Cell Neurosci..

[32] Hausburg M.A., Banton K.L., Roman P.E., Salgado F., Baek P., Waxman M.J., Tanner A., Yoder J., Bar-Or D. (2020). Effects of Propofol on Ischemia-Reperfusion and Traumatic Brain Injury. J. Crit. Care.

[33] Paracino R., De Domenico P., DI Rienzo A., Dobran M. (2024). Radiological and Blood Markers Predicting Long Term Neurological Outcome Following Decompressive Craniectomy for Malignant Ischemic Stroke: A Preliminary Single Center Study. J. Neurol. Surg. A Cent. Eur. Neurosurg..

[34] Carney N., Totten A.M., O’Reilly C., Ullman J.S., Hawryluk G.W.J., Bell M.J., Bratton S.L., Chesnut R., Harris O.A., Kissoon N. (2017). Guidelines for the Management of Severe Traumatic Brain Injury, Fourth Edition. Neurosurgery.

[35] Bergold P.J. (2016). Treatment of Traumatic Brain Injury with Anti-Inflammatory Drugs. Exp. Neurol..

[36] Kalra S., Malik R., Singh G., Bhatia S., Al-Harrasi A., Mohan S., Albratty M., Albarrati A., Tambuwala M.M. (2022). Pathogenesis and Management of Traumatic Brain Injury (TBI): Role of Neuroinflammation and Anti-Inflammatory Drugs. Inflammopharmacology.

[37] Brawanski A. (2012). On the Myth of the Edwin Smith Papyrus: Is It Magic or Science?. Acta Neurochir..

